# Identification of diagnostic markers for MINOCA in ST-segment elevation myocardial infarction patients

**DOI:** 10.3389/fcvm.2025.1690879

**Published:** 2025-11-18

**Authors:** John Michael Hoppe, Michael Christoph Schramm, Kathrin Diegruber, David Esser, Steffen Massberg, Christopher Stremmel

**Affiliations:** 1 Department of Medicine IV, LMU University Hospital, Munich, Germany; 2 Department of Medicine I, LMU University Hospital, Munich, Germany; 3 DZHK (German Centre for Cardiovascular Research), Partner Site Munich Heart Alliance, LMU University Hospital, Munich, Germany

**Keywords:** MINOCA, ST-segment elevation myocardial infarction, myocarditis, Takotsubo syndrome, acute coronary syndrome

## Abstract

**Introduction:**

Coronary artery disease remains the leading cause of death globally, with ST-segment elevation myocardial infarction (STEMI) requiring immediate intervention. However, some STEMI patients are later diagnosed with myocardial infarction with non-obstructive coronary arteries (MINOCA). Differentiating MINOCA is challenging and often hampered by limited access to advanced imaging. This study examines MINOCA patient characteristics and explores whether demographics, routine laboratory, and ECG findings can help differentiate MINOCA subgroups in the absence of advanced imaging.

**Methods:**

We conducted a retrospective single-center study of 2,553 suspected consecutive STEMI cases between 2013 and 2023. After excluding acute obstructive coronary artery disease and missing data, 296 patients were analyzed based on final diagnosis and compared by clinical, laboratory and diagnostic characteristics.

**Results:**

Among 296 patients, 205 (69.3%) met MINOCA criteria. Coronary causes (9.1%) included embolism and plaque rupture. Cardiac non-coronary causes (47.6%) included (peri-) myocarditis, non-STEMI (NSTEMI) type 2, and Takotsubo cardiomyopathy. Non-cardiac causes (5.4%), such as pulmonary embolism and aortic dissection, were less common. NSTEMI type 1 occurred in 3.7%, and 27.0% had no identifiable cause.

Patients with (peri-) myocarditis were significantly younger, had lower BMI, higher CK and CRP levels, and more frequent ST-segment elevations. In contrast, NSTEMI type 2 patients were older, more often in shock, had more comorbidities, and used cardiovascular medications more frequently.

**Conclusion:**

In the absence of advanced imaging, routine clinical and laboratory parameters can provide critical information to differentiate MINOCA subtypes and guide the urgency of downstream diagnostic tests. In resource-limited settings, they could provide a framework for future risk-based scoring systems to optimize imaging use and improve patient care.

## 1 Introduction

Coronary artery disease (CAD) has been the leading cause of mortality worldwide for many years (1). The low ischemic tolerance of myocardial tissue highlights the critical need for prompt recognition and management of affected patients. To address this need, specialized care structures such as chest pain units have been established, employing clearly defined treatment algorithms aimed at the rapid identification and treatment of acute coronary syndromes (ACS). Particularly in patients presenting with ST-segment elevation myocardial infarction (STEMI), immediate intervention is essential to minimize morbidity and mortality (2).

Despite advances in diagnostic and therapeutic strategies, a notable proportion of patients presenting with STEMI do not exhibit significant coronary artery stenoses (>50%) on angiography. These patients are classified under the working diagnosis of myocardial infarction with non-obstructive coronary arteries (MINOCA), which accounts for approximately 1%–14% of all ACS presentations (2, 3). Recognizing the clinical importance of this entity, the latest 2023 European Society of Cardiology (ESC) guidelines for ACS devote a dedicated chapter to MINOCA (2).

Identifying the underlying pathologies responsible for MINOCA remains a major diagnostic challenge. Importantly, patients with MINOCA have a considerable 12-month mortality rate of approximately 5% (3), emphasizing the necessity of accurate and timely diagnosis. Underlying causes can be broadly categorized into three groups: (I) Coronary causes including plaque rupture, coronary artery dissection, coronary embolism, vasospasm, myocardial bridging and microvascular dysfunction; (II) cardiac non-coronary causes including myocarditis, Takotsubo cardiomyopathy, other cardiomyopathies, cardiotoxicity, trauma and heart transplant graft failure; and (III) non-cardiac causes such as ARDS, allergic reactions, systemic inflammation or sepsis, pulmonary embolism, aortic dissection, stroke, renal failure and severe hypertension (2).

Cardiac magnetic resonance imaging (CMR) can establish a diagnosis in approximately 87% of MINOCA cases and has been granted a Class IB recommendation in the 2023 ESC guidelines (2). However, in real-world clinical practice, CMR remains underutilized, mainly due to limited availability, costs, and logistic constraints. Large registry data indicate global utilization rates well below 50%, and when CMR is performed, it is often delayed substantially, with a median time to CMR of about 180 days (4, 5). Consequently, although CMR represents the diagnostic gold standard, it frequently cannot contribute to acute diagnostic decision-making or risk stratification in daily practice. Therefore, the identification of MINOCA sub-entities based on routine biomarkers and ECG parameters remains of high clinical relevance.

Among laboratory markers, cardiac troponins are the most studied biomarkers for distinguishing underlying etiologies. A short-term coronary occlusion with spontaneous resolution, such as seen in embolism, spasm, or dissection, typically results in troponin elevations >5–10 times the upper reference limit (URL), whereas lower levels (1–5× URL) are more suggestive of myocarditis or Takotsubo cardiomyopathy. The average troponin T level on admission in MINOCA patients was 0.48 µg/L (6). However, a study on 50 women with MINOCA by Reynolds et al. demonstrated that maximum troponin levels are not suitable to distinguish between the different pathologies and highlighted that rather troponin dynamics might be helpful (7). This is in line with an investigation on 49 patients with suspected STEMI and MINOCA, which revealed that the troponin dynamics and natriuretic peptide levels could aid in differentiation: patients with myocarditis showed less dynamic troponin kinetics, while those with Takotsubo cardiomyopathy demonstrated relatively low troponin levels despite significant wall motion abnormalities. Moreover, elevated levels of C-reactive protein (CRP) and leukocytes on admission were associated with perimyocarditis, albeit the authors were unable to supply defined cutoff values (6).

Of note, this latter study by Stensaeth et al. also highlighted the role of N-terminal prohormone of brain natriuretic peptide (NT-proBNP) in the light of MINOCA differentiation. While patients with coronary pathologies or myocarditis usually present with lower NT-proBNP values, Takotsubo patients are characterized by unexpectedly high NT-proBNP levels—especially in relation to the extent of myocardial injury reflected by troponin quantification (6).

The D-dimer assay, traditionally used in the diagnosis of pulmonary embolism, has also been investigated in the context of MINOCA. A retrospective study of 322 STEMI patients without significant coronary stenoses identified D-dimer as a useful trigger for further evaluation for aortic dissection, with a suggested cutoff value of 750 ng/mL (8).

The largest studies on MINOCA patients in the setting of ACS showed variable distributions regarding the underlying pathologies. While the final diagnosis remains unclear in about one third of all cases, the three most common diagnoses include peri-myocarditis (50%–60%), coronary pathologies mostly identified by intravascular imaging (10%–40%) and Takotsubo cardiomyopathies (∼10%) (6, 7, 9).

Collectively, existing studies highlight the diagnostic yield of CMR, intravascular imaging and the potential utility of laboratory and ECG parameters in identifying underlying causes in MINOCA patients. Yet, many questions remain regarding how best to stratify these patients in real-world settings, where advanced diagnostics are not always feasible. Thus, the present study retrospectively evaluates whether laboratory and ECG findings can effectively differentiate underlying pathologies among STEMI patients with MINOCA, aiming to optimize diagnostic strategies when advanced imaging modalities are unavailable.

## 2 Methods

### 2.1 Study design and population

This retrospective single-center observational study was conducted at Ludwig-Maximilians-Universität (LMU) Hospital in Munich, Germany. Data were extracted from the LMU STEMI database, encompassing patients admitted between 2013 and 2023. Inclusion criteria were an initial diagnosis of STEMI upon referral or admission and the performance of coronary angiography.

**Figure 1:**
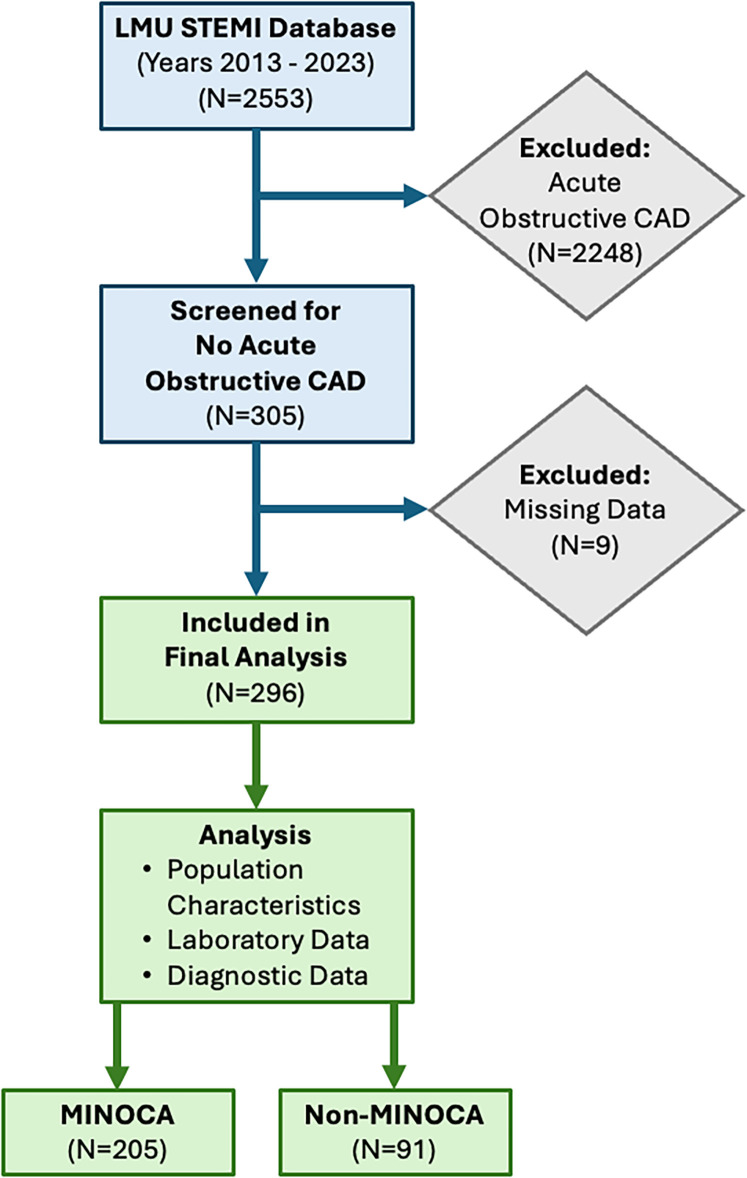
Diagram illustrating patient selection from the LMU STEMI database (2013–2023). Out of *N* = 2553 patients who underwent coronary angiography, *N* = 305 cases with no acute obstructive coronary artery disease (CAD) were identified. After excluding *N* = 9 patients due to missing data, *N* = 296 patients were included in the final anylsis assessing population characteristics, laboratory data, and diagnostic data. *N* = 205 patients were identified as having myocardial infarction with nonobstructive coronary arteries (MINOCA), while *N* = 91 patients were classified as non-MINOCA.

For this analysis, we screened all patients in whom acute obstructive CAD (defined as ≥50% stenosis) was excluded as the underlying cause of ST-segment elevation or clinical symptoms. Among 2,553 patients who underwent angiography, 305 cases were identified with no acute obstructive CAD. After excluding 9 cases due to incomplete clinical or diagnostic data, 296 patients were included in the final analysis (Figure 1).

### 2.2 MINOCA diagnostic work-up

Patients were stratified by final diagnosis into MINOCA subgroups, including coronary, non-coronary cardiac, and non-cardiac causes. Clinical characteristics, laboratory parameters, and diagnostic findings were systematically compared across these groups.

The diagnostic work-up of MINOCA cases was performed at the discretion of the attending physician and followed local clinical standards. In almost all patients, a structured medical history, physical examination, 12-lead resting ECG, and coronary angiography were performed upon presentation. If coronary angiography revealed no hemodynamically significant stenosis, a left ventriculography was routinely performed during the same procedure to assess wall-motion abnormalities and ejection fraction.

The indication for further diagnostic testing—such as intravascular imaging (IVUS or OCT), vasospasm provocation testing or non-invasive modalities performed outside the catheterization laboratory (e.g., CMR, computed tomography, long-term ECG monitoring, or transesophageal echocardiography)—was based on the clinical judgment of the attending physician and individual patient presentation.

Vasospasm testing was not performed systematically. In selected cases with angiographically apparent transient vessel narrowing and compatible ischemic symptoms or ECG changes, intracoronary nitroglycerin was administered to confirm reversibility. Formal pharmacologic provocation with intracoronary acetylcholine was not part of the acute diagnostic protocol, in line with current ESC recommendations, which state that such testing may be considered in patients with suspected vasospastic angina but is not routinely necessary in the acute ACS setting due to the small but relevant risk of complications (2). Cases in which vasospasm could not be confidently confirmed were conservatively classified as “*unknown/other.”*

All diagnostic data were reviewed retrospectively for the present analysis, and the final diagnosis was adjudicated by two independent cardiologists.

### 2.3 ST-segment elevation

All patients included in this study were initially classified as STEMI cases based on the diagnosis made at first medical contact. In each case, the initial ECG findings were independently reviewed by experienced cardiologists who were blinded to the final diagnosis. Any discrepancies among the experts were resolved by consensus*.* ST-segment elevations were re-evaluated and re-classified according to the criteria defined in the most recent ESC guidelines for the management of acute coronary syndromes (2).

### 2.4 Ethical approval

The study was conducted in accordance with the Declaration of Helsinki and approved by the local institutional ethics committee (LMU Munich; approval number #23-0609). Owing to the retrospective nature of the study, the requirement for informed consent was waived.

### 2.5 Statistical analysis

Statistical analyses were performed using Prism 10 software (GraphPad, USA). Population characteristics were reported as median and inter quartile range (IQR). Group comparisons were conducted using mean, standard deviation and two-tailed unpaired t test for continuous variables. For categorical variables, proportions were compared using Fisher's exact test. *P* values < 0.05 were considered statistically significant. Subsequently, the diagnostic performance of the five best-fitting variables, selected based on highest statistical significance (lowest *p*-values) and a prevalence of at least 10% in either cohort, was evaluated using univariate ROC curve analysis. In addition, ROC curve analysis was performed on a multiple logistic regression model incorporating the combination of variables demonstrating the highest diagnostic performance. No prior sample size calculation was performed.

## 3 Results

### 3.1 Study population

A total of 296 patients initially admitted with suspected STEMI, but in whom acute obstructive CAD was later excluded based on coronary angiography, were included in the analysis. Among these, 205 patients (69.3%) were diagnosed with MINOCA, including coronary causes such as coronary embolism (3.7%), plaque rupture (2.7%), coronary vasospasm (2.0%), and spontaneous coronary artery dissection (SCAD) (0.7%). Non-coronary cardiac causes included (peri-) myocarditis (19.9%), non-ST-segment elevation myocardial infarction (NSTEMI) type 2 (11.8%), Takotsubo cardiomyopathy (5.4%), other cardiomyopathies (3.7%), perioperative ST-segment elevation (3.7%) and arrhythmias (3.0%). Non-cardiac causes were pulmonary embolism (2.7%), aortic dissection (1.4%), and sepsis (1.4%). An additional 7.1% were attributed to other MINOCA-related causes, such as hypertension and myocardial bridging. Non-MINOCA cases included NSTEMI type 1 (3.7%) and in 80 patients (27.0%) either no pathology was identified, or the underlying cause remained unknown. Detailed information on demographics, clinical characteristics, laboratory findings, medical history, diagnostic tests and medication is presented in Table 1.

**Table 1:** Demographics, clinical characteristics and diagnostic findings.

Characteristic	Total	MINOCA, *N* = 205 (69.3%)														NSTEMI Type 1	No or unknown pathology
		Coronary causes, *N* = 27 (9.1%)				Non-coronary, cardiac causes, *N* = 141 (47.6%)						Non-cardiac causes, *N* = 16 (5.4%)			Other		
		Coronary embolism	Plaque rupture	Coronary vasospasm	SCAD	(Peri-) myocarditis	NSTEMI Type 2	Takotsubo syndrome	Cardiomyopathy	Perioperative	Arrhythmia	Pulmonary embolism	Aortic dissection	Sepsis			
*N*	296 (100.0%)	11 (3.7%)	8 (2.7%)	6 (2.0%)	2 (0.7%)	59 (19.9%)	35 (11.8%)	16 (5.4%)	11 (3.7%)	11 (3.7%)	9 (3.0%)	8 (2.7%)	4 (1.4%)	4 (1.4%)	21 (7.1%)	11 (3.7%)	80 (27.0%)
Clinical parameters																	
Age (Years)	61.0 (47.1; 73.9)	62.0 (52.5; 76.9)	51.9 (47.3; 59.3)	59.5 (55.1; 73.0)	46.0 (44.1; 47.9)	46.3 (32.0; 61.7)	70.8 (60.5; 76.0)	76.7 (68.0; 79.9)	57.7 (39.9; 69.0)	68.4 (65.7; 75.1)	64.5 (50.2; 75.0)	68.3 (58.9; 78.6)	67.6 (52.6; 77.3)	67.8 (57.8; 74.7)	61.9 (54.6; 72.5)	72.5 (62.7; 81.2)	55.1 (45.0; 69.6)
Sex																	
Male	217 (73.3%)	5 (45.5%)	7 (87.5%)	5 (83.3%)	1 (50.0%)	46 (78.0%)	25 (71.4%)	3 (18.8%)	9 (81.8%)	6 (54.5%)	5 (55.6%)	7 (87.5%)	3 (75.0%)	3 (75.0%)	16 (76.2%)	9 (81.8%)	67 (83.8%)
Female	79 (26.7%)	6 (54.5%)	1 (12.5%)	1 (16.7%)	1 (50.0%)	13 (22.0%)	10 (28.6%)	13 (81.2%)	2 (18.2%)	5 (45.5%)	4 (44.4%)	1 (12.5%)	1 (25.0%)	1 (25.0%)	5 (23.8%)	2 (18.2%)	13 (16.2%)
Weight (kg)	80.0 (70.0; 86.0)	75.0 (65.0; 85.0)	80.0 (78.8; 86.3)	72.5 (61.3; 80.0)	82.5 (81.3; 83.8)	75.0 (69.0; 85.0)	80.0 (69.0; 83,5)	69.0 (60.5; 80.0)	80.0 (73.5; 92.5)	75.0 (74.9; 90.0)	78.0 (79.0; 91.0)	80.0 (70.0; 82.5)	72.5 (70.0; 86.3)	74.0 (66.0; 83.8)	80.0 (75.0; 85.0)	80.0 (76.0; 82.5)	79.0 (73.5; 90.0)
Height (cm)	175 (170; 180)	165 (165; 175)	179 (174; 181)	177 (171; 180)	175 (173; 178)	180 (171; 183)	170 (170; 175)	166 (160; 170)	178 (171; 182)	170 (164; 176.5)	176 (168; 180)	171 (170; 174)	175 (169; 183)	173 (169; 176)	172 (165; 180)	176 (167; 180)	178 (172; 183)
BMI (kg/m^2^)	25.1 (23.4; 27.7)	25.7 (24.2; 28.5)	26.4 (24.7; 26.6)	23.6 (20.8; 24.7)	27.1 (25.9; 28.2)	24.5 (23.2; 27.6)	25.3 (24.2; 27.4)	24.4 (23.0; 27.5)	25.7 (24.7; 28.9)	26.5 (25.5; 32.9)	24.7 (24.4; 28.1)	26.7 (25.1; 27.7)	25.0 (24.0; 27.4)	25.5 (23.9; 26.9)	26.2 (24.7; 29.4)	25.0 (24.3; 28.3)	25.5 (24.0; 27.7)
Ejection Fraction (%)	60 (45; 60)	48 (39; 60)	48 (33; 59)	60 (60; 60)	50 (48; 53)	60 (45; 60)	60 (48; 60)	40 (30; 40)	45 (38; 60)	60 (40; 60)	60 (60; 60)	60 (60; 60)	60 (60; 60)	60 (60; 60)	60 (43; 60)	40 (37; 47)	60 (60; 60)
Shock	50 (16.9%)	4 (36.4%)	1 (12.5%)	0 (0.0%)	0 (0.0%)	4 (6.8%)	11 (31.4%)	2 (12.5%)	2 (18.2%)	3 (27.3%)	1 (11.1%)	2 (25.0%)	3 (75.0%)	3 (75.0%)	9 (42.9%)	3 (27.3%)	2 (2.5%)
ECMO	9 (3.0%)	2 (18.2%)	0 (0.0%)	0 (0.0%)	0 (0.0%)	1 (1.7%)	1 (2.9%)	0 (0.0%)	0 (0.0%)	3 (27.3%)	0 (0.0%)	0 (0.0%)	2 (50.0%)	0 (0.0%)	0 (0.0%)	0 (0.0%)	0 (0.0%)
CPR	11 (3.7%)	1 (9.1%)	0 (0.0%)	0 (0.0%)	0 (0.0%)	0 (0.0%)	2 (5.7%)	1 (6.3%)	0 (0.0%)	0 (0.0%)	0 (0.0%)	0 (0.0%)	1 (25.0%)	0 (0.0%)	3 (14.3%)	2 (18.2%)	1 (1.3%)
ECG findings																	
Significant ST-Elevation	109 (36.8%)	7 (63.6%)	8 (100.0%)	4 (66.7%)	2 (100.0%)	40 (67.8%)	6 (17.1%)	7 (43.8%)	5 (45.5%)	4 (36.4%)	1 (11.1%)	1 (12.5%)	3 (75.0%)	1 (25.0%)	7 (33.3%)	0 (0.0%)	13 (16.3%)
Osborn Wave	12 (4.2%)	0 (0.0%)	0 (0.0%)	0 (0.0%)	0 (0.0%)	4 (6.8%)	1 (2.9%)	0 (0.0%)	0 (0.0%)	1 (9.1%)	0 (0.0%)	0 (0.0%)	0 (0.0%)	0 (0.0%)	2 (9.5%)	0 (0.0%)	4 (5.0%)
LBBB	16 (5.5%)	0 (0.0%)	0 (0.0%)	0 (0.0%)	0 (0.0%)	1 (1.7%)	1 (2.9%)	1 (6.3%)	2 (18.2%)	0 (0.0%)	0 (0.0%)	2 (25.0%)	1 (25.0%)	0 (0.0%)	0 (0.0%)	2 (18.2%)	6 (7.5%)
RBBB	11 (3.7%)	0 (0.0%)	0 (0.0%)	0 (0.0%)	0 (0.0%)	1 (1.7%)	1 (2.9%)	0 (0.0%)	0 (0.0%)	0 (0.0%)	1 (11.1%)	0 (0.0%)	0 (0.0%)	0 (0.0%)	3 (14.3%)	0 (0.0%)	5 (6.3%)
Medication																	
ASA	95 (32.1%)	3 (27.3%)	3 (37.5%)	1 (16.7%)	1 (50.0%)	7 (11.9%)	23 (65.7%)	2 (12.5%)	3 (27.3%)	5 (45.5%)	4 (44.4%)	2 (25.0%)	1 (25.0%)	1 (25.0%)	8 (38.1%)	7 (63.6%)	24 (30.0%)
Clopidogrel	18 (6.1%)	3 (27.3%)	1 (12.5%)	2 (33.3%)	1 (50.0%)	0 (0.0%)	4 (11.4%)	0 (0.0%)	0 (0.0%)	1 (9.1%)	0 (0.0%)	1 (12.5%)	0 (0.0%)	0 (0.0%)	1 (4.8%)	3 (27.3%)	1 (1.3%)
Ticagrelor	2 (0.7%)	0 (0.0%)	0 (0.0%)	0 (0.0%)	0 (0.0%)	0 (0.0%)	1 (2.9%)	0 (0.0%)	0 (0.0%)	0 (0.0%)	0 (0.0%)	0 (0.0%)	0 (0.0%)	0 (0.0%)	1 (4.8%)	0 (0.0%)	0 (0.0%)
Prasugrel	10 (3.4%)	0 (0.0%)	2 (25.0%)	0 (0.0%)	0 (0.0%)	1 (1.7%)	4 (11.4%)	0 (0.0%)	0 (0.0%)	0 (0.0%)	0 (0.0%)	0 (0.0%)	0 (0.0%)	0 (0.0%)	0 (0.0%)	2 (18.2%)	1 (1.3%)
Phenprocoumon	7 (2.4%)	2 (18.2%)	0 (0.0%)	0 (0.0%)	0 (0.0%)	1 (1.7%)	1 (2.9%)	2 (12.5%)	0 (0.0%)	0 (0.0%)	0 (0.0%)	0 (0.0%)	0 (0.0%)	0 (0.0%)	0 (0.0%)	0 (0.0%)	1 (1.3%)
NOAC	26 (8.8%)	0 (0.0%)	1 (12.5%)	1 (16.7%)	0 (0.0%)	5 (8.5%)	3 (8.6%)	1 (6.3%)	0 (0.0%)	0 (0.0%)	0 (0.0%)	0 (0.0%)	1 (25.0%)	0 (0.0%)	2 (9.5%)	3 (27.3%)	9 (11.3%)
Beta Blocker	92 (31.1%)	6 (54.5%)	3 (37.5%)	1 (16.7%)	1 (50.0%)	9 (15.3%)	19 (54.3%)	5 (31.3%)	4 (36.4%)	2 (18.2%)	3 (33.3%)	1 (12.5%)	2 (50.0%)	0 (0.0%)	7 (33.3%)	7 (63.6%)	22 (27.5%)
ACEi/ARB	105 (35.5%)	3 (27.3%)	4 (50.0%)	3 (50.0%)	1 (50.0%)	10 (16.9%)	16 (45.7%)	7 (43.8%)	3 (27.3%)	3 (27.3%)	5 (55.6%)	4 (50.0%)	2 (50.0%)	1 (25.0%)	9 (42.9%)	6 (54.5%)	28 (35.0%)
Aldosteron Antagonist	19 (6.4%)	1 (9.1%)	1 (12.5%)	0 (0.0%)	0 (0.0%)	1 (1.7%)	2 (5.7%)	1 (6.3%)	3 (27.3%)	0 (0.0%)	0 (0.0%)	1 (12.5%)	0 (0.0%)	0 (0.0%)	2 (9.5%)	2 (18.2%)	5 (6.3%)
Diuretic	50 (16.9%)	4 (36.4%)	1 (12.5%)	0 (0.0%)	0 (0.0%)	6 (10.2%)	10 (28.6%)	4 (25.0%)	1 (9.1%)	0 (0.0%)	4 (44.4%)	1 (12.5%)	0 (0.0%)	2 (50.0%)	4 (19.0%)	5 (45.5%)	8 (10.0%)
Sacubitril/Valsartan	4 (1.4%)	0 (0.0%)	0 (0.0%)	0 (0.0%)	0 (0.0%)	0 (0.0%)	0 (0.0%)	0 (0.0%)	1 (9.1%)	0 (0.0%)	0 (0.0%)	0 (0.0%)	0 (0.0%)	0 (0.0%)	0 (0.0%)	1 (9.1%)	2 (2.5%)
SGLT2 Inhibitor	12 (4.1%)	0 (0.0%)	1 (12.5%)	0 (0.0%)	0 (0.0%)	2 (3.4%)	2 (5.7%)	0 (0.0%)	1 (9.1%)	0 (0.0%)	0 (0.0%)	0 (0.0%)	0 (0.0%)	0 (0.0%)	0 (0.0%)	4 (36.4%)	2 (2.5%)
Medical history																	
Myocardial Infarction	38 (12.8%)	3 (27.3%)	1 (12.5%)	2 (33.3%)	0 (0.0%)	1 (1.7%)	7 (20.0%)	2 (12.5%)	1 (9.1%)	2 (18.2%)	1 (11.1%)	1 (12.5%)	0 (0.0%)	0 (0.0%)	4 (19.0%)	5 (45.5%)	8 (10.0%)
Coronary Angiography	84 (28.4%)	4 (36.4%)	1 (12.5%)	2 (33.3%)	0 (0.0%)	6 (10.2%)	13 (37.2%)	1 (6.3%)	4 (36.4%)	8 (72.7%)	2 (22.2%)	2 (25.0%)	0 (0.0%)	1 (25.0%)	9 (42.9%)	8 (72.7%)	23 (28.8%)
Coronary Intervention	42 (14.2%)	2 (18.2%)	1 (12.5%)	1 (16.7%)	0 (0.0%)	1 (1.7%)	10 (28.6%)	0 (0.0%)	1 (9.1%)	1 (9.1%)	1 (11.1%)	2 (25.0%)	0 (0.0%)	0 (0.0%)	7 (33.3%)	6 (54.5%)	9 (11.3%)
Bypass	8 (2.7%)	0 (0.0%)	0 (0.0%)	0 (0.0%)	0 (0.0%)	0 (0.0%)	0 (0.0%)	0 (0.0%)	0 (0.0%)	7 (63.6%)	0 (0.0%)	0 (0.0%)	0 (0.0%)	0 (0.0%)	1 (4.8%)	0 (0.0%)	0 (0.0%)
Hypertension	140 (47.3%)	6 (54.5%)	2 (25.0%)	4 (66.7%)	1 (50.0%)	11 (18.6%)	22 (62.9%)	9 (56.3%)	4 (36.4%)	7 (63.6%)	6 (66.7%)	4 (50.0%)	4 (100.0%)	1 (25.0%)	14 (66.7%)	10 (90.9%)	35 (43.8%)
Hypercholesterolemia	57 (19.3%)	3 (27.3%)	0 (0.0%)	1 (16.7%)	0 (0.0%)	6 (10.2%)	9 (25.7%)	1 (6.3%)	1 (9.1%)	4 (36.4%)	1 (11.1%)	2 (25.0%)	1 (25.0%)	0 (0.0%)	7 (33.3%)	8 (72.7%)	13 (16.3%)
Diabetes	40 (13.5%)	1 (9.1%)	0 (0.0%)	1 (16.7%)	0 (0.0%)	5 (8.5%)	8 (22.9%)	0 (0.0%)	2 (18.2%)	1 (9.1%)	1 (11.1%)	1 (12.5%)	0 (0.0%)	2 (50.0%)	4 (19.0%)	3 (27.3%)	11 (13.8%)
Positive Family History	31 (10.5%)	2 (18.2%)	1 (12.5%)	1 (16.7%)	0 (0.0%)	7 (11.9%)	1 (2.9%)	0 (0.0%)	1 (9.1%)	0 (0.0%)	0 (0.0%)	0 (0.0%)	0 (0.0%)	0 (0.0%)	2 (9.5%)	1 (9.1%)	15 (18.8%)
Nicotine	59 (19.9%)	5 (45.5%)	4 (50.0%)	2 (33.3%)	2 (100.0%)	10 (16.9%)	5 (14.3%)	0 (0.0%)	3 (27.3%)	0 (0.0%)	2 (22.2%)	1 (12.5%)	1 (25.0%)	1 (25.0%)	5 (23.8%)	3 (27.3%)	15 (18.8%)
Cardiac diagnostics																	
CT Angiography	89 (30.1%)	5 (45.5%)	0 (0.0%)	1 (16.7%)	2 (100.0%)	19 (32.2%)	14 (40.0%)	3 (18.8%)	3 (27.3%)	0 (0.0%)	0 (0.0%)	8 (100.0%)	3 (75.0%)	3 (75.0%)	8 (38.1%)	1 (9.1%)	19 (23.8%)
CMR	69 (23.3%)	3 (27.3%)	1 (12.5%)	2 (33.3%)	2 (100.0%)	38 (64.4%)	6 (17.1%)	4 (25.0%)	6 (54.5%)	0 (0.0%)	0 (0.0%)	0 (0.0%)	0 (0.0%)	1 (25.0%)	1 (4.8%)	0 (0.0%)	5 (6.3%)
Long Term ECG	44 (14.9%)	2 (18.2%)	2 (25.0%)	1 (16.7%)	0 (0.0%)	14 (23.7%)	4 (11.4%)	3 (18.8%)	5 (45.5%)	0 (0.0%)	3 (33.3%)	0 (0.0%)	0 (0.0%)	0 (0.0%)	0 (0.0%)	1 (9.1%)	9 (11.3%)
TEE	23 (7.8%)	4 (36.4%)	0 (0.0%)	2 (33.3%)	1 (50.0%)	5 (8.5%)	5 (14.3%)	2 (12.5%)	1 (9.1%)	0 (0.0%)	1 (11.1%)	0 (0.0%)	0 (0.0%)	0 (0.0%)	0 (0.0%)	1 (9.1%)	1 (1.3%)
Laboratory findings																	
CK [adm] (U/L)	157 (87; 395)	145 (101; 369)	221 (123; 1,280)	87 (85; 186)	184 (134; 233)	357 (150; 752)	170 (94; 411)	212 (115; 257)	118 (82; 240)	906 (629; 1,256)	72 (54; 89)	57 (27; 101)	128 (93; 239)	178 (130; 787)	207 (105; 509)	189 (93; 273)	115 (77; 171)
CK [max] (U/L)	229 (107; 707)	690 (236; 1,191)	1,913 (791; 3,309)	173 (87; 261)	2,069 (1,199; 2,940)	495 (228; 972)	307 (153; 597)	234 (142; 419)	154 (93; 468)	1,680 (1,005; 5,402)	92 (67; 126)	77 (32; 209)	1,154 (398; 1,840)	1,354 (130; 2,680)	312 (112; 717)	431 (156; 1,041)	125 (83; 185)
CK-MB [adm] (U/L)	31 (17; 64)	126 (98; 164)	95 (22; 290)	17 (13; 31)	19 (17; 20)	40 (21; 73)	40 (26; 51)	36 (32; 42)	28 (16; 69)	74 (46; 112)	12 (11; 12)	22 (18; 27)	42 (31; 53)	40 (27; 52)	68 (26; 88)	30 (28; 38)	16 (14; 23)
CK-MB [max] (U/L)	40 (21; 91)	129 (102; 232)	317 (188; 409)	28 (18; 38)	280 (162; 397)	45 (24; 91)	41 (26; 60)	44 (36; 54)	24 (17; 98)	138 (88; 291)	13 (12; 18)	37 (25; 50)	51 (36; 58)	121 (111; 131)	80 (32; 107)	48 (35; 91)	19 (15;23)
hs-TnT [adm] (ng/mL)	0.054 (0.013; 0.552)	1.125 (0.310; 1.875)	0.100 (0.022; 1.150)	0.075 (0.022; 0.131)	0.163 (0.114; 0.211)	0.337 (0.036; 1.375)	0.200 (0.128; 0.737)	0.505 (0.240; 0.973)	0.021 (0.013; 0.040)	2.590 (0.769; 4.810)	0.021 (0.013; 0.022)	0.027 (0.016; 0.064)	0.032 (0.024; 0.102)	0.121 (0.022; 0.266)	0.041 (0.013; 0.358)	0.408 (0.166; 1.710)	0.013 (0.013; 0.017)
hs-TnT [max] (ng/mL)	0.170 (0.018; 1.270)	2.175 (0.739; 4.570)	3.980 (1.810; 8.830)	0.149 (0.040; 0.513)	0.529 (0.529; 0.529)	0.560 (0.074; 1.710)	0.504 (0.233; 1.690)	0.934 (0.521; 1.411)	0.031 (0.013; 0.053)	5.700 (3.595; 24.050)	0.021 (0.013; 0.028)	0.062 (0.023; 0.133)	0.169 (0.030; 0.549)	0.247 (0.063; 0.958)	0.049 (0.013; 0.517)	1.110 (0.529; 1.725)	0.013 (0.013; 0.024)
Creatinine [adm] (mg/dl)	1.0 (0.8; 1.2)	1.1 (0.8; 1.3)	0.9 (0.8; 1.0)	1.1 (0.9; 1.1)	0.8 (0.7; 0.9)	0.9 (0.8; 1.0)	1.1 (0.8; 1.4)	0.9 (0.8; 1.1)	1.0 (0.8; 1.1)	1.0 (0.9; 1.0)	1.0 (0.8; 1.1)	1.1 (0.8; 1.5)	1.1 (1.0; 1.2)	1.7 (1.4; 2.1)	1.2 (1,0; 1.4)	1.3 (1.0; 2.0)	1.0 (0.8; 1.1)
Creatinine [max] (mg/dl)	1.0 (0.9; 1.2)	1.1 (0.9; 1.3)	1.0 (0.9; 1.1)	1.1 (1.1; 1.1)	0.9 (0.8; 0.9)	1.0 (0.9; 1.1)	1.2 (1.0; 1.5)	1.0 (0.9; 1.1)	1.0 (0.9; 1.1)	1.2 (1.0; 1.8)	1.0 (0.9; 1.2)	1.2 (0.8; 1.5)	1.3 (1.1; 1.6)	2.3 (1.4; 3.6)	1.3 (1.0; 1.6)	1.4 (1.0; 2.1)	1.0 (0.9; 1.2)
LDH [adm] (U/L)	247 (189; 361)	394 (241; 415)	202 (181; 426)	215 (168; 363)	211 (203; 218)	263 (200; 353)	286 (235; 375)	222 (184; 408)	257 (177; 126)	416 (403; 763)	210 (203; 268)	268 (229; 513)	288 (275; 333)	516 (425; 702)	266 (214; 315)	259 (205; 361)	192 (170; 241)
LDH [max] (U/L)	267 (199; 392)	390 (283; 482)	439 (204; 724)	312 (202; 397)	421 (339; 504)	273 (211; 362)	293 (242; 435)	264 (218; 399)	319 (177; 485)	434 (408; 660)	210 (203; 271)	271 (239; 502)	394 (313; 544)	1,296 (990; 1,712)	284 (216; 405)	321 (259; 361)	197 (172; 241)
CRP [adm] (mg/dl)	0.7 (0.1; 5.3)	1.0 (0.3; 5.4)	0.4 (0.3; 0.8)	0.8 (0.2; 5.5)	0.1 (0.1; 0.1)	3.4 (1.1; 10.8)	0.7 (0.2; 6.3)	0.6 (0.3; 6.7)	0.2 (0.1; 0.6)	1.4 (0.5; 6.5)	0.5 (0.2; 1.6)	0.9 (0.5; 2.4)	0.3 (0.2; 0.4)	19.1 (17.6; 21.4)	0.9 (0.2; 5.0)	2.0 (0.4; 3.2)	0.2 (0.1; 1.0)
Leukocytes [adm] (G/L)	10.2 (7.7; 13.6)	9.8 (7.1; 12.3)	14.2 (11.8; 14.5)	9.5 (9.4; 10.4)	14.9 (10.9; 19.0)	10.4 (8.3; 11.9)	10.0 (8.8; 14.9)	13.1 (8.1; 17.0)	8.7 (7.1; 12.8)	11.6 (8.6; 14.2)	11.0 (9.1; 12.1)	13.4 (9.3; 21.3)	10.5 (9.0; 11.0)	14.9 (11.3; 18.1)	12.4 (8.1; 14.5)	9.7 (5.8; 11.8)	8.7 (6.9; 11.9)
LDL (mg/dl)	108 (73; 131)	87 (55; 125)	127 (123; 139)	ND	94 (81; 106)	99 (66; 118)	81 (76; 121)	84 (65; 106)	116 (110; 127)	110 (101; 128)	148 (140; 158)	85 (72; 99)	134 (109; 158)	43 (35; 52)	58 (53; 69)	81 (40; 105)	126 (96; 137)
TG (mg/dl)	112 (81; 157)	98 (82; 158)	102 (59; 157)	ND	61 (48; 73)	85 (72; 128)	112 (79; 157)	112 (102; 127)	118 (76; 146)	97 (90; 306)	155 (119; 184)	92 (70; 116)	114 (114; 114)	79 (69; 88)	137 (102; 165)	150 (114; 192)	124 (95; 200)
HbA1c (%)	5.6 (5.4; 6.2)	5.7 (5.4; 6.0)	5.6 (5.4; 5.6)	ND	ND	5.4 (5.3; 5.8)	5.8 (5.4; 6.5)	5.5 (5.3; 5.7)	5.7 (5.4; 6.0)	ND	ND	5.8 (5.8; 5.8)	5.5 (5.5; 5.5)	6.5 (6.5; 6.5)	6.7 (5.7; 7.3)	5.8 (5.4; 6.2)	5.9 (5.6; 6.4)
Lp(a) (nmol/L)	22 (12; 61)	17 (11; 52)	53 (32; 83)	ND	91 (91; 91)	12 (12; 22)	12 (12; 22)	17 (12; 22)	8 (8; 9)	172 (161; 182)	ND	86 (86; 86)	ND	34 (34; 34)	12 (12; 12)	18 (12; 23)	22 (12; 31)
NT-proBNP (ng/L)	514 (87; 2,120)	5,705 (3,046; 11,461)	ND	ND	871 (871; 871)	532 (256; 1,266)	354 (292; 2,531)	4,275 (660; 10,012)	99 (34; 568)	ND	35,294 (17,941; 52,647)	31,668 (31,668; 31,668)	2,023 (2,023; 2,023)	7,613 (4,849; 10,378)	4,197 (2,110; 6,285)	ND	76 (25; 208)
D-Dimer (µg/mL)	0.6 (0.5; 3.6)	1.0 (0.6; 18.4)	0.5 (0.4; 0.5)	ND	0.5 (0.5; 0.5)	0.9 (0.4; 1.8)	4.6 (1.5; 7.7)	1.2 (0.5; 1.9)	0.7 (0.5, 2.4)	1.1 (1.0; 5.4)	ND	4.3 (2.4; 7.7)	5.6 (4.0; 13.4)	15.7 (10.4; 33.1)	1.1 (0.5; 8.8)	17.6 (11.2; 23.9)	0.5 (0.4; 0.5)

Data are median (IQR) or *n* (%). MINOCA, myocardial infarction with nonobstructive coronary artery disease; SCAD, spontaneous coronary artery dissection; NSTEMI, non-ST-elevation myocardial infarction; BMI, body mass index; ECMO, extracorporeal membrane oxygenation; CPR, cardiopulmonary resuscitation; ECG, electrocardiogram; LBBB, left bundle branch block; RBBB, right bundle branch block; ASA, acetylsalicylic acid; NOAC, new oral anticoagulants; ACEi, angiotensin-converting enzyme inhibitor; ARB, angiotensin II receptor blocker; TEE, transesophageal echocardiography; CT, computed tomography; CMR, cardiac magnetic resonance imaging; hs-TnT, high sensitive troponin T; LDH, lactate dehydrogenase; CRP, C-reactive protein; CK, creatine kinase; [adm], at admission; [max], peak value; TG, triglycerides; NT-proBNP, N-terminal prohormone of brain natriuretic peptide; ND, no data.

### 3.2 Demographics and clinical characteristics

**Figure 2:**
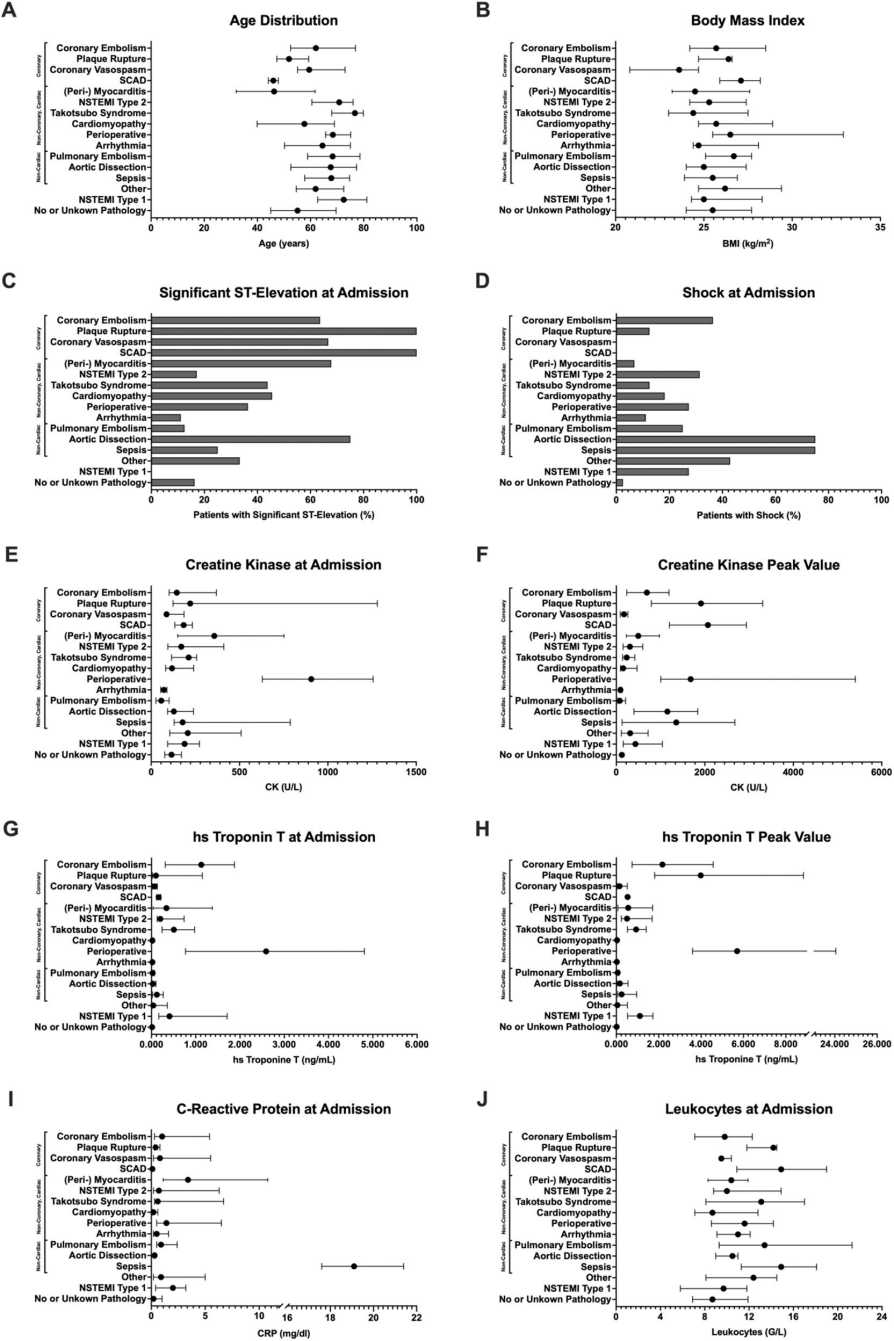
Graphical presentation of all no acute obstructive coronary artery disease patient groups for the following parameters: **(A)** age distribution, **(B)** body mass index, **(C)** presence of significant ST-elevation at admission, **(D)** shock at admission, **(E)** creatine kinase (CK) levels at admission, **(F)** CK peak value, **(G)** high-sensitivity troponin T (hs-TnT) at admission, **(H)** hs-TnT peak value, **(I)** C-reactive protein at admission, and **(J)** leukocyte count at admission. Data for **(A,B,E,F–J)** are presented as median (IQR); **(C,D)** show percentage.

The median age was 61.0 years (IQR 47.1; 73.9), with (peri-) myocarditis patients being younger (46.3 years) compared to those with Takotsubo cardiomyopathy (76.7 years) and NSTEMI type 1 (72.5 years) (Figure 2A). The majority of the cohort was male (73.3%), though certain subgroups, such as Takotsubo cardiomyopathy patients, exhibited a predominance of female individuals (81.2%). Median BMI across the cohort was 25.1 kg/m^2^ (IQR 23.4; 27.7), with no notable differences between subgroups (Figure 2B). Left ventricular ejection fraction was preserved in most groups, with a median of 60% (IQR 45; 60), except for Takotsubo cardiomyopathy and NSTEMI type 1 patients who demonstrated a notably lower ejection fraction (median 40%). Significant ST-segment elevation on ECG was present in 36.8% of patients, particularly frequent in plaque rupture (100%), SCAD (100%), aortic dissection (75%), (peri-) myocarditis (67.8%), coronary vasospasm (66.7%) and coronary embolism (63.6%) (Figure 2C). Cardiogenic shock was observed in 16.9% of all patients, highest among those with aortic dissection (75%) and sepsis (75%) (Figure 2D). Mechanical circulatory support with extracorporeal membrane oxygenation (ECMO) was employed in 3.0% of cases, predominantly in patients with aortic dissection (50%) and perioperative ST-segment elevation (27.3%). Cardiopulmonary resuscitation (CPR) was performed in 3.7% of patients, primarily among those with aortic dissection (25%) and NSTEMI type 1 (18.2%). (Table 1)

### 3.3 Laboratory findings

Median peak levels of creatine kinase (CK) and high-sensitive troponin T (hs-TnT) were 229 U/L (IQR 107; 707) and 0.170 ng/mL (IQR 0.018; 1.270), respectively. The highest CK levels were observed in patients with SCAD (2,069 U/L), plaque rupture (1,913 U/L), and perioperative ST-segment elevation (1,680 U/L) (Figure 2F). In contrast, the highest CK levels at admission were found in patients with perioperative ST-segment elevation (906 U/L) and (peri-) myocarditis (357 U/L) (Figure 2E). The highest hs-TnT admission and peak values were noted in cases of perioperative ST-segment elevation (2.590 ng/mL and 5.700 ng/mL) and coronary embolism (1.125 ng/mL and 2.175 ng/mL). Cases with plaque rupture had the most notable hs-TnT dynamic (0.100 ng/mL at admission and 3.980 ng/ml peak value) (Figures 2G,H). Inflammatory markers, such as CRP, were highest in sepsis (19.1 mg/dl) and myocarditis (3.4 mg/dl) (Figure 2I), while leukocyte count was highest in cases of sepsis and SCAD (both 14.9 G/L) (Figure 2J). Median D-dimer levels were markedly elevated in cases of NSTEMI type 1 (17.6 µg/mL), sepsis (15.7 µg/ml), aortic dissection (5.6 µg/mL), NSTEMI type 2 (4.6 µg/mL), and pulmonary embolism (4.3 µg/mL) (Table 1). However, the median D-Dimer for NSTEMI type 1 may be skewed, as only a few measurements were available for analysis.

### 3.4 Medical history, medication usage and diagnostic testing

Hypertension was the most prevalent comorbidity (47.3%), particularly in patients with aortic dissection (100%) and NSTEMI type 1 (90.9%). Antiplatelet therapy with acetylsalicylic acid (ASA) was prescribed in 32.1% of patients, most frequently in NSTEMI type 2 (65.7%) and NSTEMI type 1 (63.6%). Beta-blockers (31.1%) and angiotensin-converting enzyme inhibitors (ACEi) or angiotensin II receptor blockers (ARB) (35.5%) were also commonly administered, reflecting standard secondary prevention measures. Diagnostic imaging utilization varied by etiology. CMR was most frequently performed in (peri-) myocarditis patients (64.4%), while CT angiography was commonly utilized in SCAD (100%), pulmonary embolism (100%), sepsis (75%), and aortic dissection (75%) (Table 1).

### 3.5 Comparison of (peri-) myocarditis with all other diagnoses

Among the 296 patients included in the analysis, 59 (19.9%) were diagnosed with peri-myocarditis. Compared to patients with other etiologies, those with (peri-) myocarditis exhibited distinct clinical, demographic, diagnostic and therapeutic characteristics.

**Figure 3:**
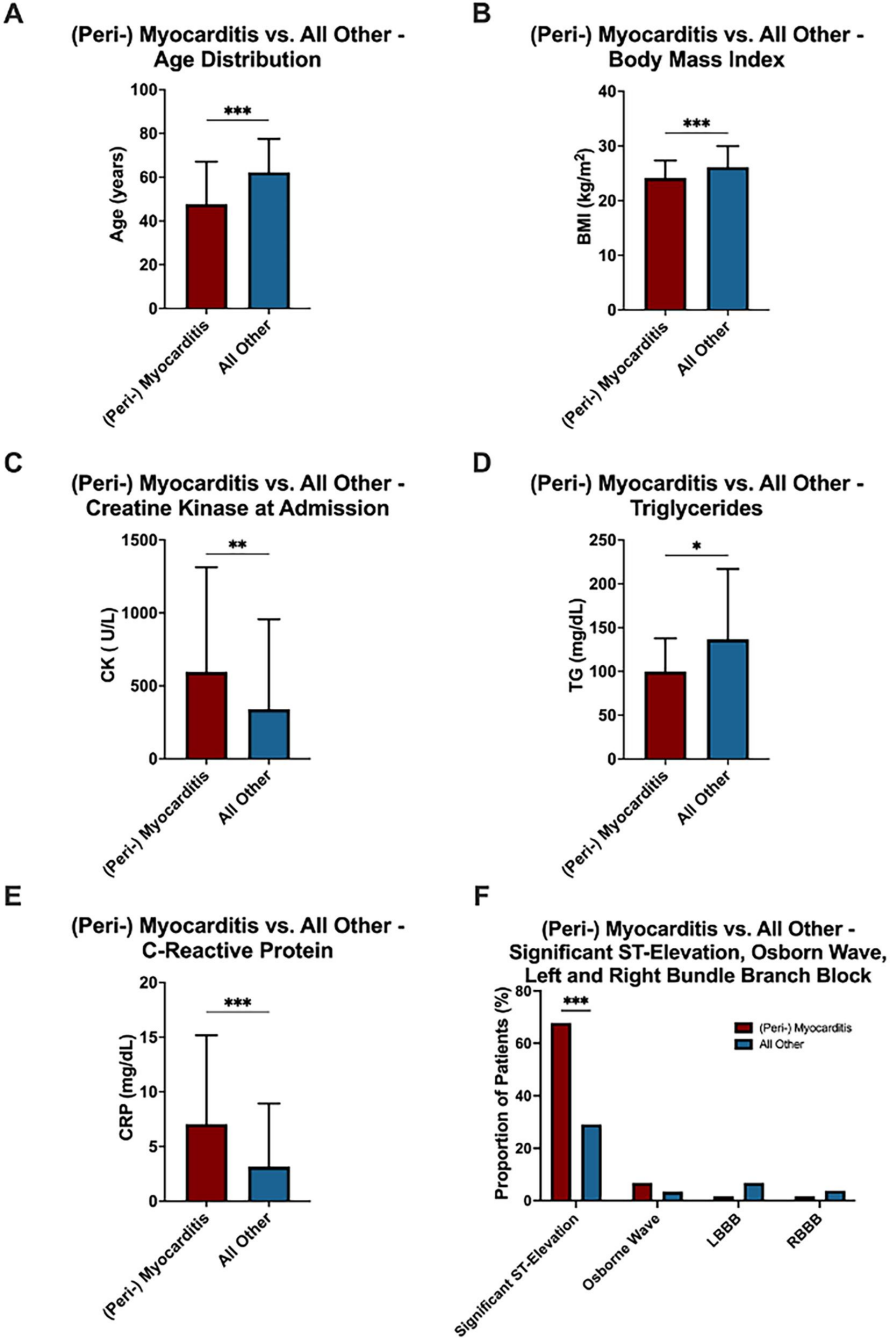
Comparison between patients with (peri-) myocarditis (*N* = 59; red) and all other patients (*N* = 237; blue) in the no acute obstructive coronary artery disease study population. **(A)** Age distribution, **(B)** BMI, **(C)** CK levels at admission, **(D)** triglycerides (TG), and **(E)** C-reaktive protein levels at admission are shown. Data in (A-E) are presented as mean ± SD. **(F)** Percentage distribution of categorical variables. Statistical significance: *p < .05; **p < .01; ***p < .001.

**Figure 4:**
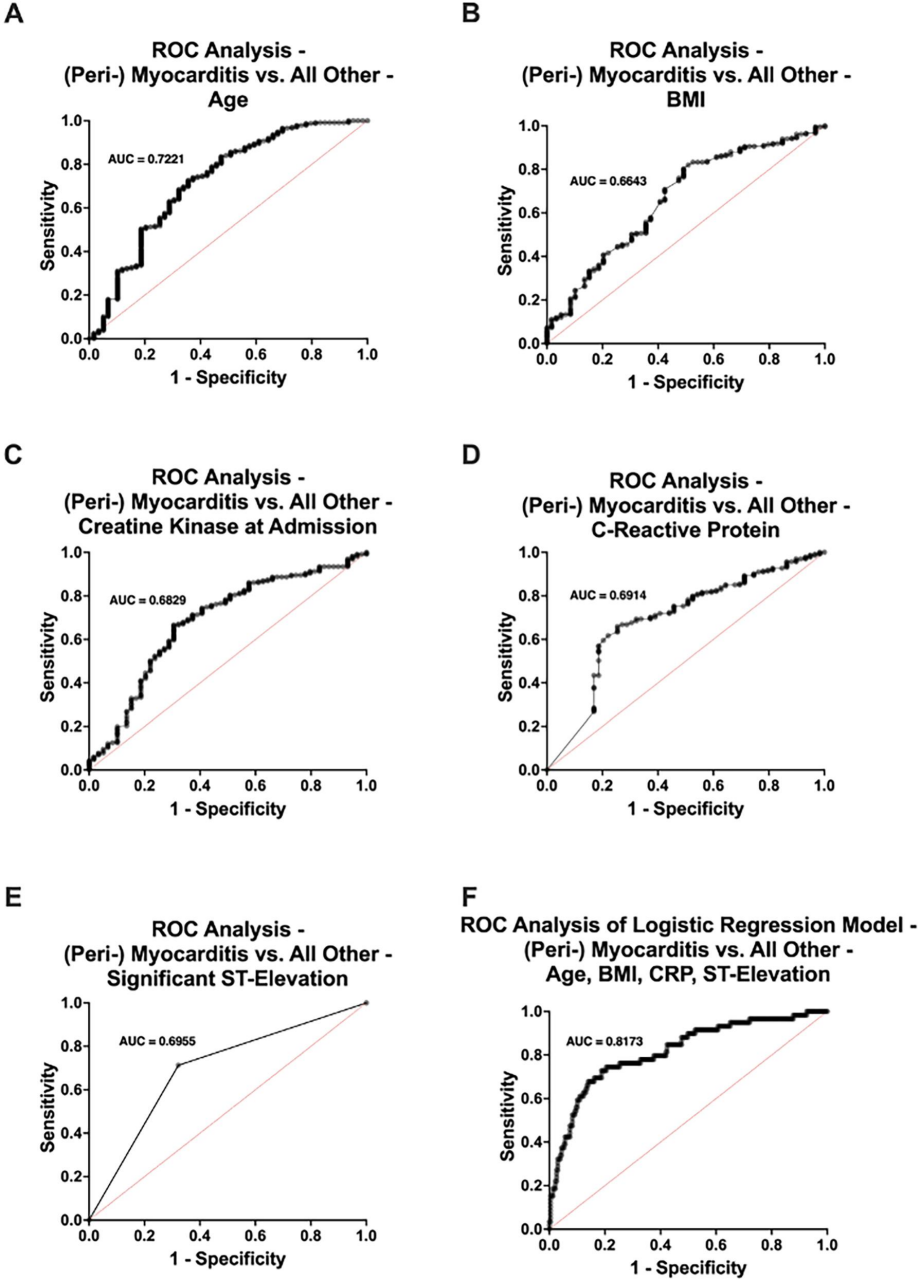
Receiver operating characteristic (ROC) curve analysis comparing patients with (peri-) myocarditis (*n* = 59) and those without (*n* = 237) in the no acute obstructive coronary artery disease study population. Univariate ROC analyses were performed for **(A)** age, **(B)** body mass index (BMI), **(C)** creatine kinase (CK) levels at admission, **(D)** C-reactive protein (CRP) levels, and **(E)** presence of significant ST-segment elevation. **(F)** Multivariable ROC curve derived from a logistic regression model including age, BMI, CRP levels, and significant ST-segment elevation. AUC, area under the curve.

Patients with (peri-) myocarditis were significantly younger (*p* < 0.001), with a mean age of 46.3 years compared to 62.2 years in the remaining cohort (Figure 3A). BMI was also significantly lower in the (peri-) myocarditis group (mean: 23.4 vs. 26.2 kg/m^2^, *p* < 0.001) (Figure 3B). (Peri-) Myocarditis patients had significantly higher elevated CK at admission compared to all other patients (mean: 595 vs. 340 U/L, *p* = 0.007) (Figure 3C). Interestingly, triglyceride (TG) levels were significantly lower in patients with (peri-) myocarditis compared to all other groups (mean: 100 vs. 137 mg/dl, *p* = 0.048), likely reflecting their younger age and lower prevalence of coronary artery disease (Figure 3D). Notably, CRP was substantially higher in (peri-) myocarditis (mean: 7.0 vs. 3.2 mg/dl, *p* < 0.001), consistent with an inflammatory pathogenesis (Figure 3E). Significant ST-segment elevation was more commonly observed in (peri-) myocarditis patients (67.8% vs. 29.1%, *p* < 0.001). Other ECG abnormalities such as LBBB and RBBB were less frequent in this group (Figure 3F). Univariate ROC curve analyses of age, BMI, CK at admission, CRP, and significant ST-segment elevation demonstrated individually modest predictive value for (peri-) myocarditis. In contrast, a multivariable logistic regression model incorporating age, BMI, CRP, and significant ST-segment elevation showed a substantially improved discriminative ability for predicting (peri-) myocarditis (AUC = 0.8173) (Figure 4).

### 3.6 Comparison of NSTEMI type 2 with all other diagnoses

Of the 296 patients analyzed, 35 (11.8%) were diagnosed with NSTEMI type 2. Compared to patients with other underlying causes, those with NSTEMI type 2 demonstrated unique clinical, demographic, diagnostic and treatment profiles.

**Figure 5:**
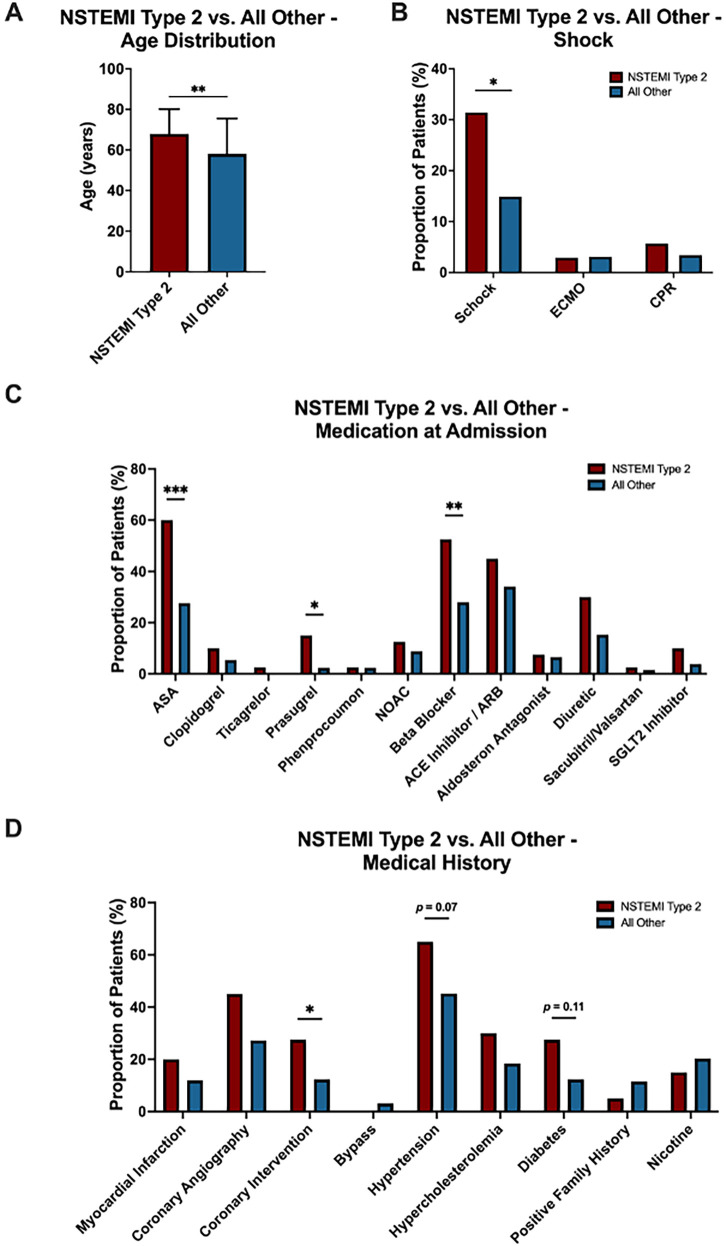
Comparison between patients with NSTEMI type 2 (*N* = 35; red) and all other patients (*N* = 261; blue) in the no acute obstructive coronary artery disease study population. **(A)** Age distribution, **(B)** proportion of patients with shock **(C)** medication at admission and **(D)** medical history are shown. Data in **(A)** is presented as mean ± SD. (B-D) Percentage distribution of categorical variables. Statistical significance: *p < .05; **p < .01; ***p < .001.

**Figure 6:**
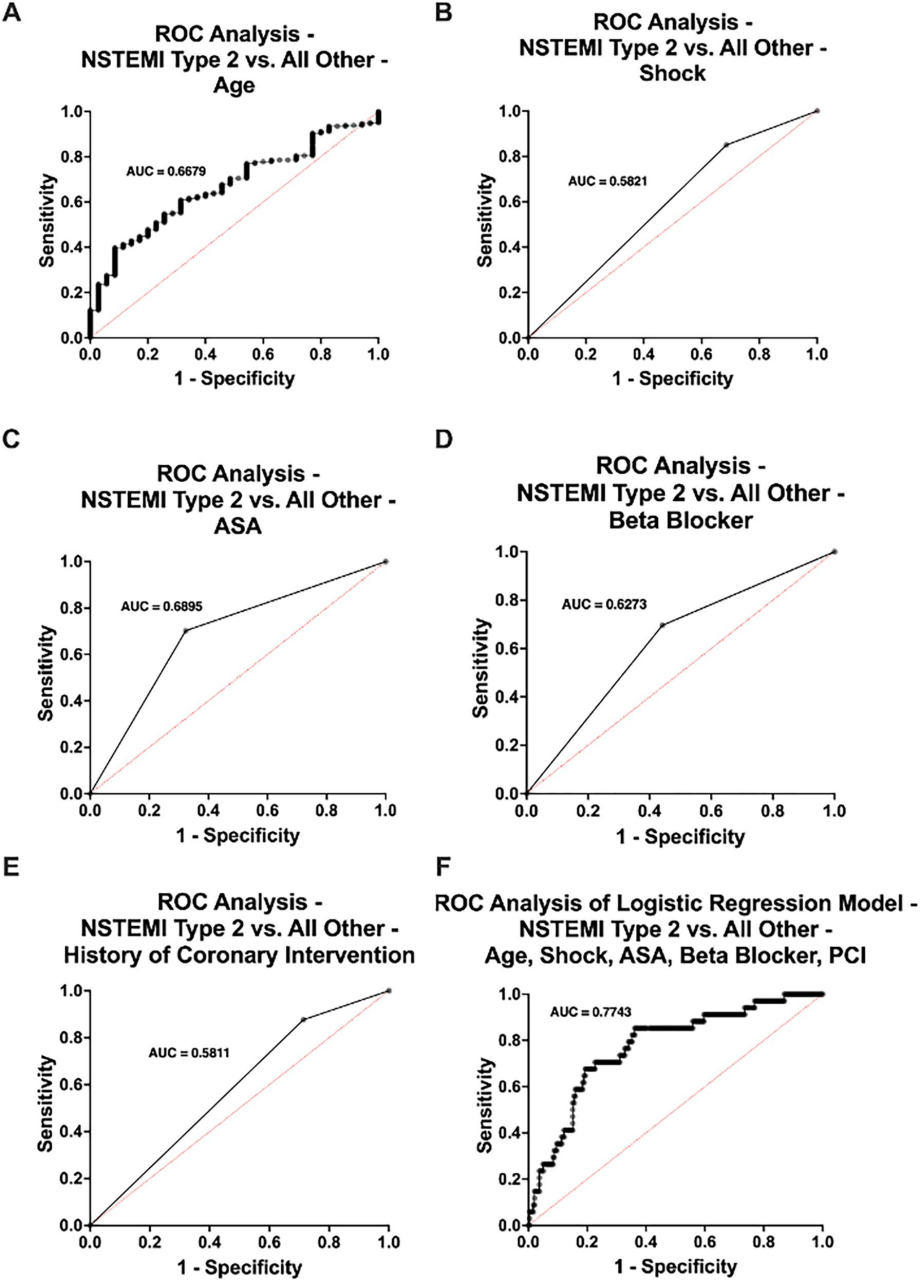
Receiver operating characteristics (ROC) curve analysis comparing patients with NSTEMI type 2 (*n* = 35) and those without (*n* = 261) in the no acute obstructive coronary artery disease study population. Univariate ROC analyses were performed for **(A)** age, **(B)** shock, **(C)** history of acetylsalicylic acid (ASA) use, **(D)** beta blocker therapy, and prior percutaneous coronary intervention (PCI). **(F)** Multivariable ROC curve derived from a logistic regression model including age, shock, history of ASA use, beta blocker therapy, and PCI. AUC, area under the curve.

Patients diagnosed with NSTEMI type 2 were significantly older (*p* = 0.001), with a mean age of 67.9 years compared to 58.1 years in the rest of the cohort (Figure 5A). Additionally, patients with NSTEMI type 2 were more likely to present with shock (31.4% vs. 14.9%, *p* = 0.027) (Figure 5B). The two groups also differed notably in their pre-admission medication profiles. Individuals with NSTEMI type 2 were significantly more likely to have been prescribed acetylsalicylic acid (ASA) (65.7% vs. 27.6%, *p* < 0.001), Prasugrel (11.4% vs. 2.3%, *p* = 0.021) and beta blockers (54.3% vs. 28.0%, *p* = 0.006) (Figure 5C). Additionally, patients with NSTEMI type 2 had a distinct medical history, showing a significantly higher prevalence of prior percutaneous coronary interventions (PCI) (28.6% vs. 12.3%, *p* = 0.039) and a trend toward more hypertension (62.9% vs. 45.2%, *p* = 0.070) and diabetes (22.9% vs. 12.3%, *p* = 0.110) compared to those with other etiologies (Figure 5D). Univariate ROC curve analyses of age, presence of shock, history of ASA use, beta blocker therapy, and prior PCI demonstrated individually modest predictive value for (peri-)myocarditis. In contrast, ROC curve analysis of a multivariable logistic regression model incorporating all these variables showed a substantially improved discriminative ability for predicting (peri-)myocarditis (AUC = 0.7743) (Figure 6).

## 4 Discussion

In this single-center retrospective study of 2,553 patients initially admitted with suspected STEMI, we found that a substantial proportion of 205 patients (8.0%) were classified as MINOCA. This proportion is in line with previous reports which estimate MINOCA to account for approximately 5%–15% of all acute myocardial infarction presentations (10–14). Importantly, we demonstrate that routine clinical and laboratory parameters, including demographics, pre-existing medical history, ECG patterns and biomarkers, offer valuable insights into the underlying etiology of MINOCA—particularly in the absence of advanced imaging modalities.

Our findings underscore the heterogeneity of MINOCA, with (peri-) myocarditis, NSTEMI type 2, and Takotsubo cardiomyopathy being the most frequent etiologies. Notably, patients with myocarditis were significantly younger, had lower BMI, higher CRP and CK levels, and more frequently presented with significant ST-segment elevations compared to other groups. These observations are consistent with previous studies suggesting a distinct inflammatory profile and highlight the potential of using basic parameters to trigger early suspicion and targeted diagnostics for myocarditis (7, 11, 15).

Importantly, the discriminative potential of these routine parameters was further supported by our ROC curve analyses: univariate analyses of age, BMI, CRP and CK levels, and significant ST-segment elevation individually demonstrated only modest predictive value for (peri-) myocarditis. However, when these variables were combined in a multivariable logistic regression model, the predictive performance improved considerably (AUC = 0.8173).

Conversely, patients with NSTEMI type 2 were significantly older, exhibited a higher burden of cardiovascular comorbidities, including hypertension and diabetes, and polypharmacy, reflecting a more typical ischemic risk profile (16, 17). These findings are in line with those of Collinson and Lindhal, who noted that NSTEMI type 2 is commonly associated with chronic illnesses and secondary to an underlying cause. In our analysis, univariate ROC curves of age, shock, history of ASA use, beta-blocker therapy, and prior PCI showed only limited predictive accuracy for NSTEMI type 2. However, when these parameters were combined in a multivariable logistic regression model, the discriminative ability improved substantially (AUC = 0.7743), underscoring the value of integrating multiple routine clinical features to enhance diagnostic predictability. This supports the idea that clinical context and prior history remain essential to differentiating type 2 infarction from other MINOCA subtypes (17).

Our results align with existing evidence that CMR substantially improves diagnostic certainty, particularly in myocarditis and Takotsubo syndrome (18). However, the reality remains that CMR is not routinely available in many acute or resource-limited settings. Our study addresses this gap by highlighting that even in the absence of advanced imaging, basic clinical and laboratory parameters can serve as surrogate indicators that may help clinicians prioritize diagnostic steps. With regard to biomarkers, CRP was significantly elevated in myocarditis and sepsis, while D-dimer was particularly useful for identifying non-cardiac causes such as pulmonary embolism and aortic dissection. These observations are consistent with previous reports and further support the use of these markers as part of a structured diagnostic algorithm for MINOCA patients (2, 7, 15).

Although only STEMI patients were included in this study, significant ST-segment elevations were not universally present. Based on the reevaluation of ECG findings by expert cardiologists according to our study protocol, only one third of our MINOCA patients presented with significant ST-segment elevations based on current ESC guidelines (2). This finding underscores the diagnostic ambiguity in the emergency setting and supports the growing recognition that the term “STEMI” may not always reflect an obstructive coronary event, particularly in MINOCA.

This study has several limitations. First, its retrospective design and single-center setting limit generalizability. Second, not all patients underwent standardized imaging (e.g., CMR performed in only 23.3%), introducing potential classification bias and therefore increasing the risk of diagnostic misclassification. However, this CMR utilization rate closely mirrors real-world data, where global CMR implementation in MINOCA remains far below 50%, often performed with substantial delay after the index event (median 180 days) due to limited availability and resources (4, 5). Third, due to the observational nature, no causal relationships can be inferred. Furthermore, the relatively high proportion of patients (36.8%) in whom significant ST-segment elevation was not confirmed after blinded ECG re-evaluation reflects a common real-world phenomenon of initial STEMI over-triage at first medical contact. Large registry analyses have demonstrated that approximately 5%–15% of pre-hospital or emergency STEMI activations are ultimately reclassified as non-STEMI or non-ischemic conditions (19, 20). Based on our total STEMI population (*n* = 2,553), this proportion would correspond to roughly 200–250 expected false-positive activations, which would—consistent with clinical experience—be overrepresented among patients without obstructive coronary artery disease, i.e., within the MINOCA subgroup. Thus, our findings realistically capture the diagnostic uncertainty inherent to acute emergency triage and reflect a representative real-world spectrum of patients presenting with suspected STEMI but unobstructed coronary arteries. Finally, the relatively small sample sizes in certain etiologies (e.g., SCAD) constrained the statistical power to identify subgroup-specific trends.

Despite these constraints, the findings show that in the absence of CMR, the current gold standard for MINOCA diagnosis, routine clinical and laboratory parameters such as age, BMI, CRP levels and ECG changes can assist in raising early clinical suspicion for specific MINOCA subtypes and guide further evaluation. These data may serve as a foundation for risk stratification tools or scoring systems, especially relevant in emergency departments, small hospitals, or other resource-limited settings where advanced imaging modalities are not readily available. Such future risk-based scoring systems could support clinical decision-making regarding the appropriate use of advanced imaging. For instance, patients with inflammatory profiles or suggestive ECG findings could be prioritized for CMR, whereas those presenting with typical ischemic patterns, but fewer red flags, might be managed with lower diagnostic urgency.

Importantly, our study does not aim to demonstrate superiority or equivalence to guideline-recommended diagnostic algorithms such as the ESC 2023 pathways, which remain the clinical gold standard for the evaluation of patients with suspected MINOCA (2). Rather, our results provide pragmatic, real-world insights into how routinely available clinical, ECG, and laboratory data can serve as a complementary decision aid in the initial triage of patients, particularly where advanced imaging (e.g., CMR or intracoronary imaging) is delayed or unavailable. In this sense, our work adds an operational perspective to guideline recommendations by highlighting that even simple, widely accessible parameters can meaningfully support diagnostic prioritization in the early phase of patient management.

Future studies should aim to validate these findings prospectively. Further, the development of clinical scoring systems that integrate clinical baseline data could enhance the efficiency, equity and timelines of care for patients with suspected MINOCA, especially where access to advanced imaging is constrained.

## Data Availability

The raw data supporting the conclusions of this article will be made available by the authors, without undue reservation.
